# Protocol for Quantitative Estimation of Hydrogen Cyanide Production from Bacteria

**DOI:** 10.21769/BioProtoc.5441

**Published:** 2025-09-20

**Authors:** Devashish Pathak, Pushpendra Sharma, Venkadasamy Govindasamy, Archna Suman

**Affiliations:** 1 Division of Microbiology, ICAR–Indian Agricultural Research Institute, New Delhi, India; 2 Department of Microbiology, Dr Rajendra Prasad Central Agricultural University, Pusa, India

**Keywords:** HCN, Potassium ferrocyanide, Secondary metabolites, Colorimetric assay, Isopurpurate

## Abstract

Hydrogen cyanide (HCN) is a volatile, nitrogen-containing secondary metabolite produced by various bacterial species, primarily during the idiophase of growth under nutrient-limiting or competitive conditions. It plays a significant ecological role as a biocontrol agent by inhibiting the respiratory enzymes of plant pathogens and modulating microbial competition in the rhizosphere. Although protocols for detecting HCN production have existed for over a century, they have largely remained qualitative and are rarely optimized for quantitative assessment. This is mainly due to the volatile nature of HCN, unidentified stable reference standards, and the absence of a robust, universally accepted protocol that ensures consistency across diverse microbial types. In this study, we present a simplified and efficient colorimetric method to quantify HCN production in both Gram-positive and Gram-negative bacteria. Qualitatively, HCN production was observed by a color change due to the isopurpurate complex. This compound was then eluted and quantified by measuring absorbance at 625 nm. The method uses potassium ferrocyanide as a standard, whose slow dissociation constant enables a stable and controlled release of cyanide ions for calibration, unlike highly dissociative salts like KCN that introduce early volatilization errors. This protocol demonstrated high sensitivity, capable of detecting HCN at concentrations as low as ppm levels, with strong correlation to the standard curve (R^2^ > 0.99). Achieving such sensitivity with other conventional methods, such as gas detection tubes or electrochemical sensors, often requires more sophisticated instrumentation and strict handling conditions. In contrast, this approach offers a cost-effective, reproducible, and user-friendly alternative. While a universally adopted method is still lacking due to standardization challenges and HCN volatility, the proposed protocol marks a significant advancement toward accurate and accessible quantitative assessment in microbiological and agricultural applications.

Key features

• Enables both qualitative detection and quantitative estimation of hydrogen cyanide (HCN) production in bacteria using a colorimetric assay.

• Utilizes a low-dissociation reference compound, potassium ferrocyanide, to create a stable and accurate standard curve for reproducible measurement of HCN concentration.

• Offers a simple, cost-effective, and broadly applicable method suitable for screening HCN-producing bacteria in both Gram-positive and Gram-negative groups.

• Highly sensitive and accurate HCN detection at sub-ppm levels, ensuring rapid colorimetrical results and reproducibility.

## Graphical overview


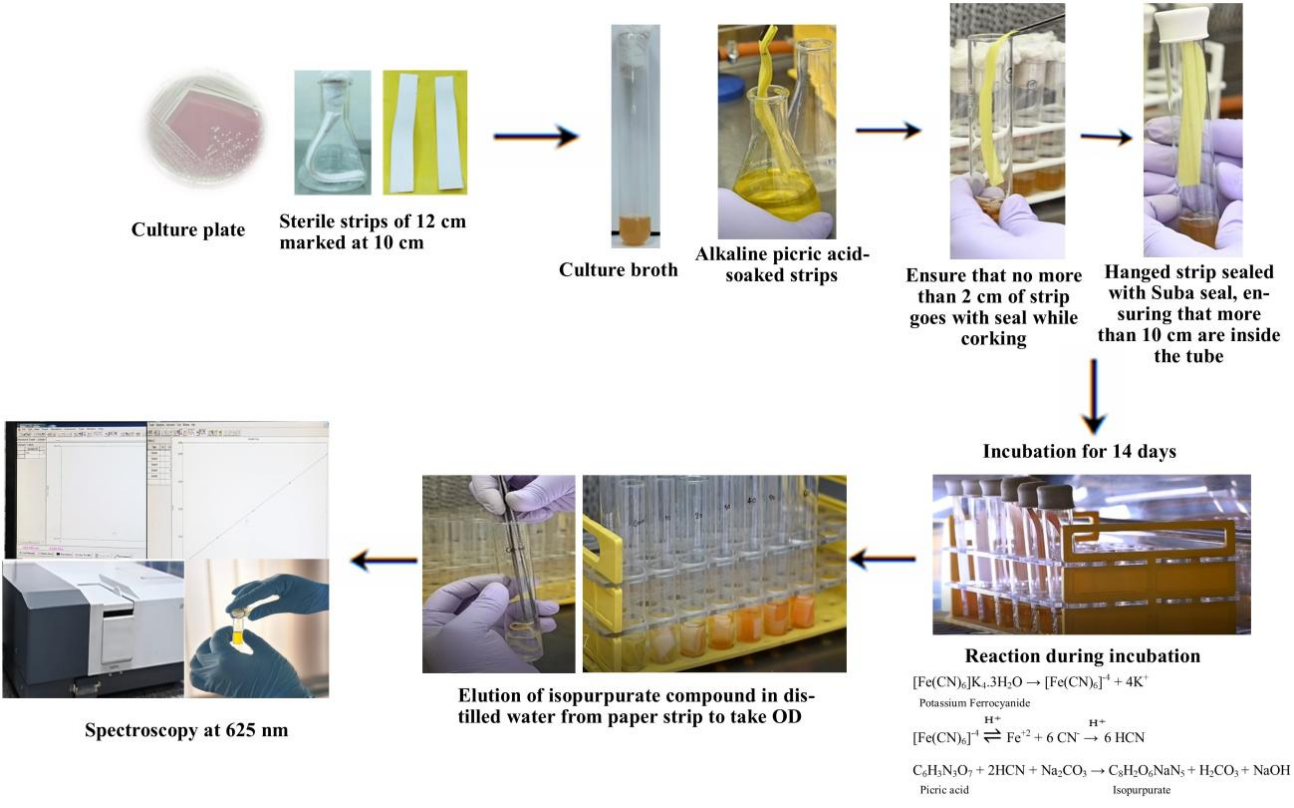


## Background

Bacteria producing HCN have plant growth–promoting (PGP) characteristics, being included in biofertilizer production as a biocontrol agent [1]. The role of HCN is to display its toxic effect on the respiration of other microbes, where cyanide binds to the metal ions present in cytochrome oxidase. Particularly, it inhibits the electron transport chain by binding with the carrier ions, i.e., Cr^6+^and Fe^3+^, and checks regular energy supply, resulting in the ultimate death of the pathogens [2]. Most of the bacterial species, mainly rhizobacteria, release HCN as secondary volatile metabolites under nutrient stress conditions in their idiophase. Major HCN producers are *Pseudomonas, Bacillus, Alkaligenes*, and *Klebsiella pneumoniae* [3]. Production of the HCN is catalyzed by a membrane-bound HCN synthase complex, encoded by the hcnABC operon, which converts glycine into HCN and carbon dioxide [4]. Biosynthesis is tightly regulated and induced under specific environmental conditions such as low oxygen availability, stationary growth phase, and the presence of glycine, highlighting its role as a competitive metabolite rather than a constitutive product [5]. Regulatory systems like the anaerobic regulator ANR, the quorum-sensing transcription factors LasR/RhlR, and the GacS/GacA two-component system play central roles in controlling hcnABC expression in response to environmental and population-level cues [6,7]. These regulatory networks integrate oxygen levels, cell density, and nutrient status to fine-tune HCN production, ensuring it occurs primarily under nutrient-limiting or competitive conditions typical of the rhizosphere. Despite being toxic, HCN is efficiently secreted extracellularly, allowing the producing bacteria to inhibit surrounding pathogens while avoiding self-harm. This well-regulated secondary metabolism confers a selective advantage in microbial communities, particularly in plant-associated niches.

Some of the experimental evidence now suggests that there is no consistent correlation between the amount of HCN produced by rhizobacteria in vitro and their biocontrol performance. Specifically, strains characterized as HCN-positive (HCN) did not exhibit strong inhibition against common phytopathogens due to HCN production. In vitro assays also revealed that the minimum inhibitory concentration (MIC) of cyanide required to impact microbial growth was approximately tenfold higher than what is naturally produced by HCN-producing bacteria, suggesting that its levels in the rhizosphere are unlikely to exert broad-spectrum antimicrobial activity [8].

Instead, an alternative functional role of HCN in rhizosphere ecology is emerging, one that centers on its capacity to mobilize essential nutrients through metal chelation. Notably, HCN forms stable complexes with divalent and trivalent transition metals, such as Fe^3+^, Al^3+^, and Ca^2+^, thereby impacting nutrient dynamics in the soil [9]. One of the most critical implications of this interaction is its effect on phosphate (PO_4_^3-^) availability. In acidic soils, phosphate readily forms insoluble complexes with iron and aluminum, limiting its accessibility to both plants and microbes [10]. In calcareous soils, similar immobilization occurs via calcium phosphate precipitation [11]. The action mechanism proposed is that HCN can bind with iron, either preventing the initial formation of insoluble Fe-PO_4_ complexes or releasing phosphate from already precipitated iron-phosphate compounds. The latter mechanism appears more favorable, as lower concentrations of cyanide (particularly KCN in experimental setups) were sufficient to liberate phosphate from mineral-bound forms. In controlled in vitro studies, the addition of potassium cyanide to granite-based mineral sand led to a measurable increase in both solution conductivity and phosphate concentration, confirming HCN’s role in mineral dissolution and phosphate mobilization.

Previously, qualitative estimation was based on a color change in the picric acid, which changes from a yellow color to reddish brown or dull pink. This color change occurs due to the formation of isopurpurate compound in the presence of HCN in an alkaline medium [12], which can be estimated for the quantification of the HCN produced. The ease, low cost, and flexibility of the colorimetric assay have made it a critical tool for the study of microbial HCN production.

## Materials and reagents

**Biological materials**

1. Gram-positive isolates: *Bacillus altitudinis, Bacillus amyloliquefaciens*, and *Bacillus haynesii*

2. Gram-negative isolates: *Pantoea agglomerans, Kosakonia cowanii*, and *Brucella intermedia*

**Reagents**

1. LuriaBertani (LB), Luria HiVeg broth, sterile powder (HiMedia, SKU: MV575G500G)

2. Tryptone (HiMedia, SKU: CR014)

3. Yeast extract (HiMedia, SKU: RM027)

4. Sodium chloride (NaCl) (HiMedia, SKU: GRM031)

5. King’s B broth base, dehydrated, King’s Medium A Base (commonly called King’s B base) (HiMedia, SKU: M1543500G)

6. Proteose peptone (HiMedia, SKU: RM005)

7. Glycerol (HiMedia, SKU: GRM081)

8. Potassium phosphate dibasic anhydrous (K_2_HPO_4_) (HiMedia, SKU: GRM3945)

9. Magnesium sulphate heptahydrate (MgSO_4_·7H_2_O) (HiMedia, SKU: PCT0008)

10. Glycine (cell culture–tested grade) (HiMedia, SKU: TC075100G)

11. Picric acid, saturated, aqueous (HiMedia, SKU: S026100ML)

12. Sodium carbonate anhydrous (Na_2_CO_3_) (HiMedia, SKU: MB253)

13. Potassium ferrocyanide (K_4_[Fe(CN)_6_]) (HiMedia, SKU: GRM1048)

14. Double-distilled water (ddH_2_O)

**Solutions**

1. Luria-Bertani (LB) broth (see Recipes)

2. King’s B broth (supplemented with glycine) (see Recipes)

3. Alkaline picrate solution (see Recipes)

4. Potassium ferrocyanide standard solutions (see Recipes)

**Recipes**

**1. Luria-Bertani (LB) broth**

**Table:** 

Reagent	Quantity or volume (per liter)
Tryptone	10 g
Yeast extract	5 g
NaCl	10 g
Distilled water	up to 1 L

Adjust pH to 7.0. Autoclave at 121°C for 15 min.

**2. King’s B broth (supplemented with glycine)**

**Table:** 

Reagent	Quantity or volume (per liter)
Proteose peptone	20 g
Glycerol	10 mL
K_2_HPO_4_	1.5 g
MgSO_4_·7H_2_O	1.5 g
Distilled water	Up to 1 L

Adjust pH to 7.0. Autoclave at 121°C for 15 min. Add filter-sterilized glycine to a final concentration of 4.4 g/L after autoclaving. Dispense 10 mL into each sterile test tube under aseptic conditions.

**3. Alkaline picrate solution**

**Table:** 

Reagent	Quantity or volume
Picric acid	2.5 g
Na_2_CO_3_	12.5 g
Double-distilled water (ddH_2_O)	Up to 1,000 mL

Mix both components thoroughly until fully dissolved. Store in a tightly closed amber bottle at room temperature.

**4. Potassium ferrocyanide standard solutions**

1. Use K_4_[Fe(CN)_6_] as a standard to prepare 0–50 ppm of CN ion equivalents.

b. Use the following molecular conversion:

422.4 g potassium ferrocyanide = 156 g of CN-

c. Prepare standards in 10 mL of ddH_2_O per tube (e.g., 0, 10, 20, 30, 40, and 50 ppm CN equivalents).

d. Cover immediately to prevent evaporation or contamination.

**Laboratory supplies**

*Note: Prepare and autoclave sufficient double-distilled water (ddH_2_O) and ensure availability of sterile test tubes, conical flasks, forceps, scissors, and pipettes.*

1. Sterile culture tubes or conical flasks (HiMedia, catalog numbers: PW1233 and LA449

2. Sterile measuring cylinders, conical flasks, and beakers (HiMedia, catalog numbers: LA454, LA449, and LA450

3. Sterile filter paper (Whatman No. 1 or equivalent), cut into 12 × 1 cm strips (HiMedia, catalog number: MB059)

*Note: For filter paper strip preparation, first cut Whatman No. 1 or equivalent filter paper into 12 × 1 cm strips, marking at 10 cm using a pencil. Sterilize strips* via *autoclaving (by keeping inside the autoclavable bag). Soak sterilized strips in alkaline picrate solution for 1–2 min. Let excess drip off in sterile conditions (e.g., under a laminar flow hood). Use immediately or store in sterile, sealed containers.*

4. Sterile Suba seal or rubber stopper (HiMedia, catalog number: LA410)

5. 0.22 μm syringe filter (HiMedia, catalog number: ME038)

6. Parafilm, (HiMedia, catalog number: PW009)

7. Sterile forceps and scissors (HiMedia, catalog numbers: LA396 and LA394)

8. Sterile pipettes and tips (HiMedia, catalog numbers: PW1149 and MBP001–MBP003)

## Equipment

1. Spectrophotometer (SHIMADZU, model: UV-3600 UV-VIS, catalog number: UV-3600-A112950)

2. Incubator (SANCO, model: B.O.D incubator JL-1028, catalog number: SAN-200)

3. Laminar airflow cabinet (JINAN BIOBASE BIOTECH, model: BBS-DDC, catalog number: BBS11V011402003D)

4. Autoclave (iGENE LABSERVE, model: LCD Vertical Autoclave, catalog number: PUR-96-LCD)

5. Orbital shaker/incubator shaker (YIHDER, model: LM-570RD, catalog number: LM-TW/2014)

6. Vortex mixer (TARSONS, model: SPINIX^TM^ Vortex Shaker, catalog number: 3020 SN 7903)

7. Computer (DELL, model: Intel^®^ Core^TM^ i5 system)

## Procedure

**A. Bacterial culture preparation**

1. Inoculate the purified bacterial strain into pre-prepared LB broth and incubate overnight at 28 ± 2 °C in a shaker incubator (150–180 rpm) to allow active growth. Maintain a blank in the same conditions.

2. Dispense 10 mL of King’s B broth (with glycine) into sterile test tubes under aseptic conditions.

3. Transfer 1% (v/v) of the overnight-grown culture into each test tube containing King’s B broth and incubate until the culture reaches an OD_600_ ≈ 1.0, indicating idiophase (secondary metabolite production phase).

**B. Paper strip handling and setup**

**Figure 1.**
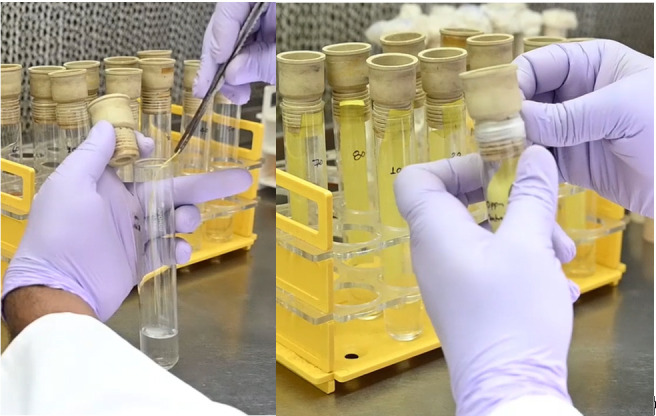
Insertion of the strip and sealing it with Suba seals and Parafilm

1. Using sterile forceps, take the alkaline picrate–soaked filter strips and hang them vertically inside each test tube, ensuring 10 cm of the strip remains inside the headspace above the bacterial broth without touching the liquid (Figure 1).

2. Seal the tubes tightly with Suba seals or rubber stoppers and further wrap the seals with Parafilm to prevent gas leakage.

3. Incubate the sealed tubes at 28 ± 2 °C for 14 days without disturbance.

**C. Standard curve assay (parallel setup)**

1. Place pre-treated alkaline picrate filter strips inside sterile test tubes containing 10 mL of potassium ferrocyanide standard solutions (ranging from 0 to 50 ppm CN- equivalents).

2. Seal and incubate these tubes under the same conditions as for the test culture (28 ± 2 °C for 14 days).

3. After incubation, carefully cut each strip at the 10 cm mark using sterile scissors and place it into 10 mL of ddH_2_O in a clean tube or beaker.

4. Gently shake or vortex the tubes to fully elute the color from the strip, forming the isopurpurate compound.

**D. Color elution from bacterial samples**

**Figure 2.**
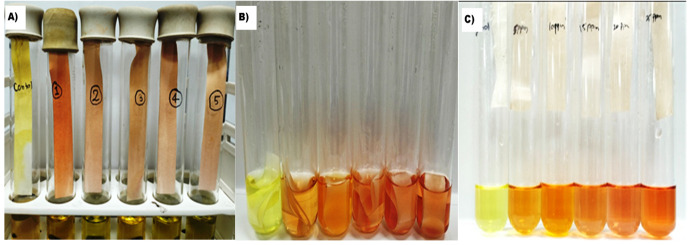
Hydrogen cyanide (HCN) production assays. (A) Color development for different isolates. (B) Color elution from filter paper at different concentrations of potassium ferrocyanide. (C) Eluted color of isopurpurate.

1. Repeat the same cutting and elution process (as in step C4) for strips recovered from bacterial culture tubes and the blank.

2. Ensure equal volume and shaking conditions are maintained for all samples (Figure 2).

**E. Measurement and quantification**

1. Measure the absorbance (OD) at 625 nm for each eluted solution using a spectrophotometer.

2. Plot a standard curve using OD values from the potassium ferrocyanide standards against known CN- concentrations.

3. Use this curve to estimate the HCN concentration in each bacterial sample by correlating its OD value.

4. Apply the derived linear regression formula (e.g., *x = y/0.0013-0.0076*) to calculate precise HCN production in ppm, where *x* = HCN concentration and *y* = OD_625_.

## Data analysis

**A. Preparation of the 50 ppm stock of CN- ion from potassium ferrocyanide**

1. The molecular weight of potassium ferrocyanide trihydrate (K_4_[Fe(CN)_6_]·3H_2_O) is approximately 422.39 g/mol.

2. Each molecule of potassium ferrocyanide contains six cyanide ions (CN).

3. The molar mass of one CN ion is 26.02 g/mol.

4. Therefore, one mole of potassium ferrocyanide provides 6 × 26.02 = 156.12 g of cyanide.

5. The proportion of cyanide in potassium ferrocyanide is 156.12/422.39 ≈ 0.3695 or 36.95%.

6. A concentration of 50 ppm corresponds to 50 mg of cyanide per liter of solution.

7. To find out how much potassium ferrocyanide is needed to provide 50 mg of cyanide, divide 50 mg by 0.3695.

8. This gives approximately 135.3 mg of potassium ferrocyanide trihydrate.

9. Therefore, dissolving 135.3 mg of potassium ferrocyanide in 1 L of water will yield a 50 ppm cyanide (CN-) solution.

**B. Quantification of the CN- ion**

**Figure 3.**
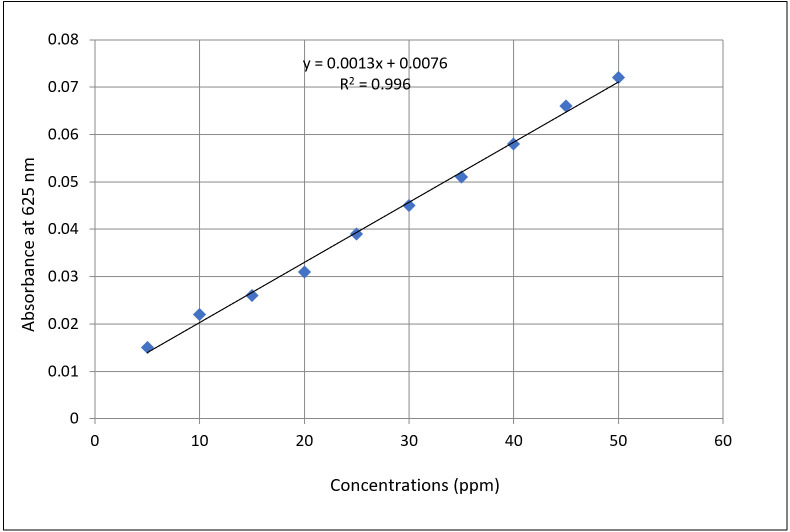
Standard curve of hydrogen cyanide (HCN) produced from potassium ferrocyanide

The quantitative estimation of hydrogen cyanide (HCN) is based on the relationship between optical density (OD) and cyanide concentration derived from a standard curve using potassium ferrocyanide (Figure 3). The standard curve equation obtained from linear regression is:

y = 0.0013x + 0.0076

Where:

y = measured absorbance at 625 nm

x = concentration of HCN (in ppm)

**C. Calculation of HCN concentration**

To determine the HCN concentration in a bacterial culture:

1. Measure the absorbance in terms of OD of the eluted isopurpurate compound from the filter strip at 625 nm.

2. Use the standard curve equation and rearrange it to solve for x:

x = y-0.00760/0013

**Example calculation:**

If the observed OD is 0.073, the calculation becomes:

x = 0.073-0.0076/0.0013

x = 0.0654/0.0013 = 50.31 ppm

The sample contains approximately 50.31 ppm of HCN.

*Note: Perform all measurements in triplicate to ensure reliability. Calculate the mean ± standard deviation for each isolate.*

## Validation of protocol

1. The absorbance values were compared with the standard curve of potassium ferrocyanide. The calculation of HCN concentration is based on the dissociation of potassium ferrocyanide. It dissociates into four K^+^ ions and one [Fe (CN)_6_]^-4^ ion, which further dissociates into six CN- ions in an aqueous condition [7].

Reaction equation:

$$[{Fe(CN)}_{6}]K_{4}\cdot 3H_{2}O\to {\left[{Fe(CN)}_{6}\right]}^{-4}+4K^{+}$$

$${\left[{Fe(CN)}_{6}\right]}^{-4}\;\overset{H^{+}}{\Leftrightarrow }{Fe}^{+2}+6{CN}^{-}\overset{H^{+}}{\to }6HCN$$

2. It was observed that two-thirds of the cyanide ions were involved in the formation of isopurpurate, while one-third went into the formation of carbonate ions [8]. This can be represented by the equation:

$$C_{6}H_{3}N_{3}O_{7}+2HCN+{Na}_{2}CO_{3}\to C_{8}H_{2}O_{6}{NaN}_{5}+H_{2}{CO}_{3}+NaOH$$

Therefore, taking OD without prior calculation of CN- ion from the standard compound would not show the real value of produced HCN.

3. The OD value obtained from the quantitative assay represents the amount of isopurpurate compound formed, which was directly related to the concentration of CN- ion calculated from potassium ferrocyanide, which represented the value of HCN produced (Table 1, Supplementary Table S1).

**Table 1.** Quantitative value of HCN production of different isolates

No.	Isolates	Organism	HCN produced (ppm)
**Gram-positive bacteria**			
1	S1	*Bacillus altitudinis*	40.24 ± 0.15
2	PC4-29	*Bacillus amyloliquefaciens*	37.90 ± 0.81
3	PHM5-9	*Bacillus haynesii*	36.78 ± 0.40
**Gram-negative bacteria**			
1	S4	*Pantoea agglomerans*	38.58 ± 0.96
2	PC4-31	*Kosakonia cowanii*	39.11 ± 0.94
3	PC4-48	*Brucella intermedia*	47.44 ± 0.49

3. The above-mentioned methodology is the next advancement in the old traditional method to quantify HCN production by bacterial isolates. This method includes the colorimetric test for the eluted color of the isopurpurate compound in double-distilled water. Through the standard curve of potassium ferrocyanide, we can get the exact concentration of cyanide for different intensities of color development. The obtained OD of eluted color with respect to the isolates can give a quantitative value of their HCN-producing ability by applying the formula x = y/0.0013 – 0.0076, where x is the concentration of the HCN, and y is the OD.

4. As HCN is a volatile compound [9], it is critical to check whether the seal was airtight or not. Any minor leakage may alter the results. For safety, it is advised to use Parafilm tape even after tightening with the cork. As in the protocol, the airtight tube may hinder the growth of aerobic bacterial isolates. To overcome this, a pre-grown culture at OD = 1 is pre-requested to take for analysis. Being a secondary metabolite, HCN production will start to be produced in the idiophase of cell growth [10], so we can give it enough time for incubation. It has been observed that as the days pass, the color of the strip starts to fade, so it is recommended to take the reading at 14 days, as this is the optimum incubation time, as proved by various trials [12,13].

5. The poor dissociation constant of potassium ferrocyanide makes this compound nearly ideal for estimating HCN production from bacterial isolates, as the slow release of the cyanide ions takes a proper incubation period. Other strong cyanide salts like KCN may cause errors in the value because of the strong dissociation constant and may release some HCN before capping of a tube. In this experiment, the dimensions of the strips were taken very precisely to avoid any error and allow equal surface area to produce the isopurpurate compound.

## General notes and troubleshooting

1. Ensure the bacterial culture reaches OD_600_ ≈ 1.0 before inserting the picrate strip to allow HCN production during the idiophase.

2. Lack of color change may indicate non-HCN-producing strains or inadequate incubation time; verify using a positive control.

3. Always suspend the picrate strip (not less than 10 cm) above the broth surface without contact to prevent smearing or underexposure.

4. Incomplete sealing with Suba seal or Parafilm may lead to HCN gas escape and false negative results.

5. Uneven or faint color development can occur due to poor strip soaking or unequal strip dimensions; maintain consistent strip preparation.

6. Elute the isopurpurate compound only after a full 14-day incubation; premature elution can cause underestimation.

7. Shake elution tubes gently but thoroughly; insufficient mixing can cause incomplete color release and variability in OD readings.

8. Use sterile, autoclaved filter paper and aseptic techniques during strip preparation and handling to avoid contamination.

9. Always calibrate the spectrophotometer before measurement and ensure cuvettes are clean and aligned correctly.

10. Prepare standard solutions freshly using potassium ferrocyanide and incubate them in identical conditions as the test samples.

11. Avoid using cyanide salts like KCN, which dissociate too quickly and can lead to inaccurate standard curves.

12. Store picric acid only in aqueous form and in tightly sealed amber bottles to prevent drying and explosion hazard.

13. Perform all steps involving picric acid and potential HCN exposure under a fume biosafety cabinet.

14. Use triplicate measurements for each sample and include both positive and negative controls in every experiment.

15. Dispose of used strips and bacterial culture broth waste according to hazardous chemical waste disposal protocols.

## Supplementary information

The following supporting information can be downloaded here:

1. Table S1: HCN production by bacterial isolates
